# The effect of school lockdown on well-being and self-esteem of adolescents during the COVID-19 pandemic in Hungary

**DOI:** 10.3389/fpubh.2024.1474893

**Published:** 2024-10-10

**Authors:** David Major, Márton Falus, Dorottya Árva, Daniel Eorsi, András Terebessy, Adam G. Tabak, Vince Fazekas-Pongor

**Affiliations:** ^1^Institute of Preventive Medicine and Public Health, Faculty of Medicine, Semmelweis University, Budapest, Hungary; ^2^Doctoral College of Semmelweis University, Budapest, Hungary; ^3^MTA-PTE Innovative Health Pedagogy Research Group, University of Pécs, Pécs, Hungary; ^4^Department of Internal Medicine and Oncology, Faculty of Medicine, Semmelweis University, Budapest, Hungary; ^5^UCL Brain Sciences, University College London, London, United Kingdom

**Keywords:** well-being, self-esteem, adolescent, COVID-19, school lockdown

## Abstract

**Introduction:**

The COVID-19 pandemic affected adolescents’ mental health diversely.

**Methods:**

Our objective was to examine the one-year change in well-being (WHO-5 well-being index) and self-esteem (Rosenberg self-esteem scale) among secondary school students affected by school lockdown (lockdown group) compared to control students unaffected by the pandemic (pre-pandemic group), utilizing data from a longitudinal survey study conducted in Hungary. We used linear mixed models stratified by sex and adjusted for family structure and family communication.

**Results:**

Two hundred twenty seven pre-pandemic (128 girls, 99 boys) and 240 lockdown (118 girls, 122 boys) students were included. Both boys’ and girls’ well-being declined in the pre-pandemic group but remained stable in the lockdown group. Post-hoc analyses on WHO-5 items revealed that the pre-pandemic and lockdown groups differed significantly on Item 4 (waking up feeling fresh and relaxed). Boys’ self-esteem did not change over the observation period in neither groups. As for girls, self-esteem of girls during lockdown increased over the observation period, while it did not change in the pre-pandemic group. Better family communication was consistently associated with higher well-being and self-esteem scores for both sexes.

**Discussion:**

Our results suggested that students may have benefitted from altered academic circumstances due to lockdown (e.g., more sleep) and students struggling with waking up early benefitted the most from lockdown (as shown in our *post hoc* analysis). Additionally, our results also indicate that families should be involved in mental health promotion interventions, especially in time of adversities. This study underscores the multifaceted effects of pandemic-related factors on adolescent mental health and highlights the need to also investigate the unexpected benefits of pandemic-related restrictions to incorporate this knowledge in health promotion programs targeting the well-being of students.

## 1 Introduction

Since 2020 when COVID-19 was declared a pandemic, a total of 703 million people were infected by Severe acute respiratory syndrome coronavirus 2 (SARS-CoV-2) and COVID-19 was responsible for 7 million deaths worldwide (1). To decrease disease burden, governments launched vaccination campaigns (2) and implemented social restrictions that severely impacted people’s life (3). Among these restrictions, school closures are notable as these restrictions forced adolescents to learn online from home and severely changed the life of affected families (4–7).

Several studies confirmed the negative impact of pandemic-related factors on adolescents’ mental health (8–10). An increasing trend of depressive symptoms, anxiety, stress and loneliness were described in the literature (8, 11). According to a systematic review of 156 observational studies, a worsening of adolescents’ well-being and self-esteem was reported in most studies during the pandemic with some reporting null-findings (12). Furthermore, it was observed that young females (age ≤ 25) were affected more harshly than males (12). The negative effects on well-being and self-esteem were explained partly by the pandemic itself (loss of control and an increase in fear, insecurity, and anger) and partly by the pandemic related social restrictions, such as school closures and social distancing that led to the social isolation of adolescents (12). Even though the majority of studies reported deteriorating mental health among the youths during the pandemic, some studies reported no change or an improvement in both well-being [defined as “the combination of feeling good and functioning well” (13)] and self-esteem [defined as “one’s perception of their own worth and value” (14)], especially at the beginning of the pandemic (15, 16).

Studies focusing on changes of well-being among adolescents often found that certain factors, such as family togetherness, better family functioning, and better quality of family relationships may counterbalance the adverse effects of the pandemic (12). Conversely, dysfunctional parenting, negative familial coping strategies, irritability of parents, and conflicts between parents and children were identified as risk factors related to worse mental health outcomes (12). For self-esteem, similar family-related factors were identified with better family relationship and communication being protective factors and familial conflict and harsh parenting being risk factors for worse self-esteem measured during the pandemic (16).

Understanding the effects of the COVID-19 pandemic on adolescents’ mental health is essential for identifying long-term psychological needs, examining the cohort effect on this generation, and improving preparedness for future public health crises. Given the low number of longitudinal studies and the mixed results regarding the association between the pandemic and well-being and self-esteem of adolescents, we aimed to compare the one-year changes in well-being and self-esteem of secondary school students affected by school lockdown to control students not affected by the pandemic using data from a longitudinal study conducted in Hungary. Our hypothesis was that well-being and self-esteem of students affected by school lockdown would show negative trends compared to the trends of control students not affected by the pandemic. As the effect of school lockdown on mental health was modulated by sex (12, 15) and family functioning (12, 17), we decided to stratify our analysis by sex and adjust for measures of family communication and family structure.

## 2 Materials and methods

### 2.1 Study design

The present study is a secondary analysis of data derived from the Balassagyarmat Health Education Program (BEP) (6, 18). BEP was a school-based health education project that focused on measuring and improving various health aspects, including sexual health, substance use, basic life support, infection control, nutrition, physical activity, and mental health of ninth-grade students from all secondary schools (three grammar schools and two vocational schools) in Balassagyarmat. Balassagyarmat is the capital of a northern Hungarian district with around 40,000 inhabitants. BEP operated between 2018 and 2021. Ninth-grade students underwent an online baseline survey before engaging in the one-year long health education program. Following the program, students completed an online follow-up survey in their 10th grade, approximately 1 year after the baseline survey.

For the current analysis, we specifically chose students with a baseline assessment in 2018 or 2020 (excluding 2019). Students with a baseline assessment in 2018 were considered the pre-pandemic group (controls) because they were not affected by the COVID-19 pandemic. This group filled in both baseline and follow-up surveys at school during teaching hours under the supervision of a research assistant without the presence of teachers. Students with a baseline assessment in 2020 were considered the lockdown group because they experienced school lockdowns due to the COVID-19 pandemic. Their baseline assessment happened similarly to the pre-pandemic group before the pandemic, the follow-up survey, however, was filled out at home during the lockdowns. Students were instructed to complete the survey during designated school hours, with the option to contact a research assistant online for any queries. We decided to exclude students enrolled in 2019 because school lockdown was undergoing an early adaptation period in Hungary at the time of their follow-up survey (March 2020). Ethical approval was obtained from the Institutional Review Board of Semmelweis University (SE TUKEB: 276/2017). Passive parental approval (opt-out) consent was sought for every participant.

### 2.2 Participants

**Figure 1:**
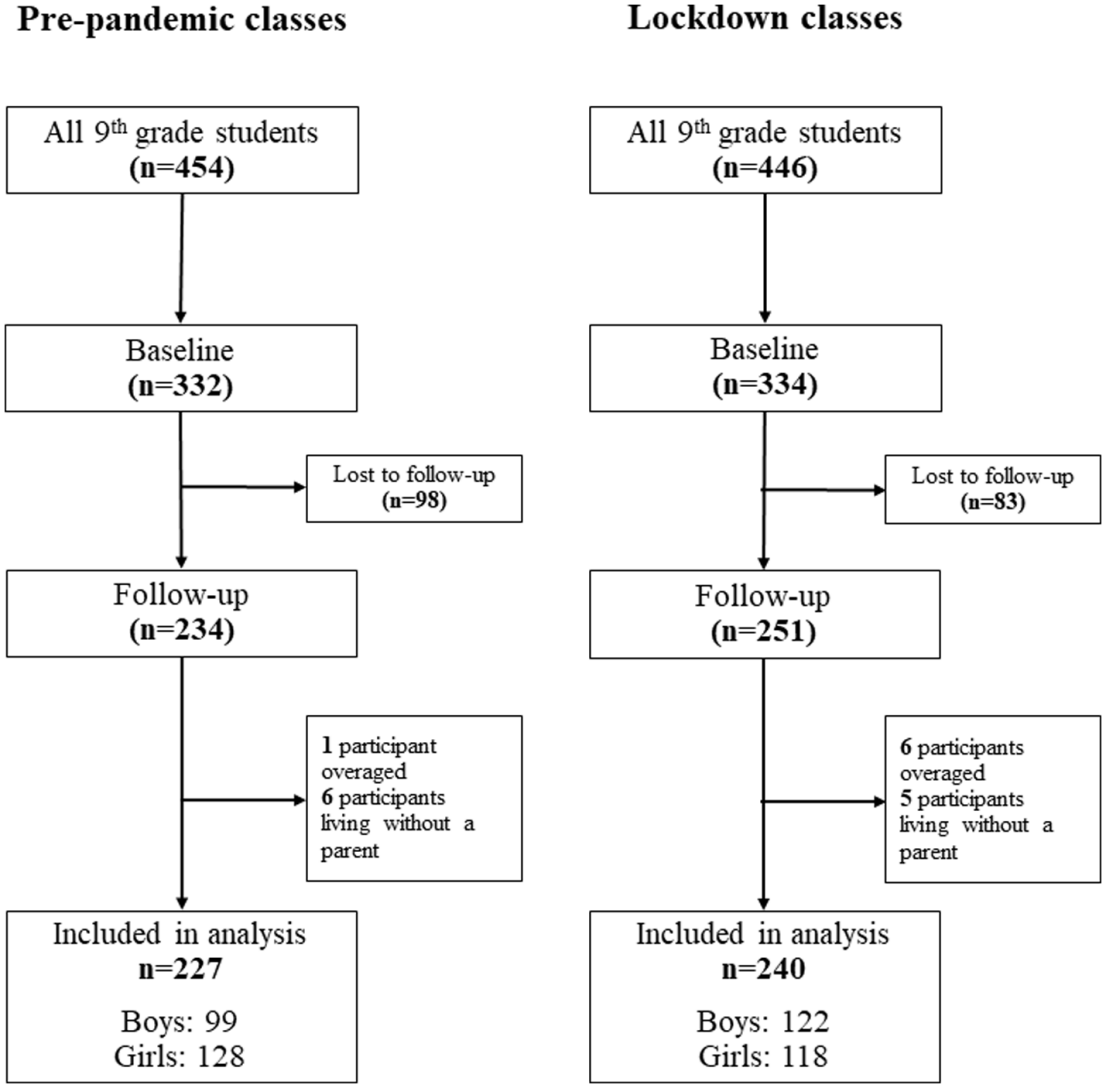
Flowchart of participants.

All ninth-graders in Balassagyarmat were invited to participate. Out of 454 eligible ninth-grade students in pre-pandemic classes, 332 (73.1%) completed the baseline survey in February 2018. Of these 98 students were lost to follow-up, thus 234 completed the follow-up survey in March 2019. As for the lockdown group, out of 446 ninth-grade students, 334 participated (74.9%) at the baseline investigation in February 2020, approximately 1 month before the COVID-19-related school lockdowns. The follow-up survey was completed by 251 students in March 2021 (Figure 1). It should be noted that all items on the full questionnaire were compulsory to fill in, so no missing individual outcomes or covariates were in the database. We further excluded overage students (mean + 3SD, *n* = 7) and participants reporting living with no parents from the analyses (*n* = 11) leaving to a final analytical sample of 227 students in the pre-pandemic group (128 girls and 99 boys) and 240 students in the lockdown group (118 girls and 122 boys) (Figure 1).

Secondary schools in Hungary were affected by the lockdowns twice during the pandemic. The first period began on 16 March 2020 and ended on 2 June 2020, while the second began on 11 November 2020 and was still ongoing at the end of our study. Excluding school holidays, public holidays, and weekends, Hungarian schools were closed for a total of 164 days between 1 January 2020 and 20 May 2021 (19).

### 2.3 Outcomes

*Well-being* was measured by the validated Hungarian version of the WHO-5 Well-being Index (WBI) (20, 21). This instrument consists of five items, each with a Likert scale ranging from 0 (at no time) to 3 points (all of the time). The items assess whether the participants (1) ‘have felt cheerful in good spirits’, (2) ‘have felt calm and relaxed’, (3) ‘have felt active and vigorous’, (4) ‘woke up feeling fresh and rested’ or (5) daily life has been filled with things that interest them in the last 2 weeks. The maximal score is 15. A higher score indicates better well-being.

*Self-esteem* was measured by the validated Hungarian version of the Rosenberg Self-esteem Scale (RSS) (14, 22), which has 10 items (5 of these with reverse scoring) with 4-point Likert scales (ranging from strongly agree to strongly disagree). The maximal score is 30. RSS is a unidimensional construct reflecting global self-esteem. A higher score is associated with higher self-esteem. The items read (1) ‘On the whole, I am satisfied with myself’; (2) ‘At times, I think I am no good at all’; (3) ‘I feel that I have a number of good qualities’; (4) ‘I am able to do things as well as most other people’; (5) ‘I feel I do not have much to be proud of’; (6) ‘I certainly feel useless at times’; (7) ‘I feel that I’m a person of worth, at least on an equal plane with others’; (8) ‘I wish I could have more respect for myself’; (9) ‘All in all, I am inclined to feel that I am a failure’; (10) ‘I take a positive attitude toward myself’.

### 2.4 Covariates

As family plays a significant role in children’s development, distress, well-being (23, 24), and self-esteem (16), our analysis was adjusted for family structure and family communication measures based on the recommendations from the Health Behavior in School-aged Children Study (25). *Family structure* was characterized by the participants’ response to the question with whom they lived together. Answers were collapsed into three categories: living with both parents, with one parent, or in a stepfamily (one biological parent and his/her new partner who lives in the same household).

*Family communication* was assessed by the Hungarian short version of the Clear Communication Scale from Family Dynamics Measure II (FDMII) (25, 26). The questionnaire has four items with a Likert scale ranging from 1 to 5 (strongly disagree to strongly agree) with a maximum score of 20. A higher score indicates a more positive assessment of family communication. The items assess whether in the family (1) ‘I think the important things are talked about’; (2) ‘When I speak someone listens to what I say’; (3) ‘We ask questions when we do not understand each other’; (4) ‘When there is misunderstanding, we talk it over until it’s clear’.

### 2.5 Statistical analysis

All analyses used a ‘full case’ design and were stratified by sex. Chi-squared tests for the categorical variable family structure and independent samples t-tests for the continuous variables (age and family communication) were used to compare baseline data of pre-pandemic vs. lockdown classes as well as follow-up data of pre-pandemic vs. lockdown classes, respectively.

Marginal homogeneity tests for the categorical variable family structure and paired t-tests for continuous variables (age and family communication) were used to compare baseline vs. follow-up data within pre-pandemic and lockdown groups.

Linear mixed models were used to assess changes associated with COVID-19-related school lockdowns in well-being (WHO-5 WBI) and self-esteem (RSS). *Model 1* includes group status (pre-pandemic/lockdown) as the sole predictor, while *Model 2* is adjusted for time-varying family structure and family communication. Both models include a random slope and a random intercept with an unstructured covariance matrix.

To further clarify the possible causes of the unexpected improvement in well-being during the pandemic, we run separate models for each question of the WHO-5 WBI using similar linear mixed models with group status as the only predictor (*Model 1*). These models showed that the only significant difference between the pre-pandemic and the lockdown groups was in the change in the score on Item 4 (waking up feeling fresh and rested) over follow-up. Thus, we hypothesized that those adolescents who struggled with waking up early profited the most from the lockdowns. To test this hypothesis, first we divided the students into two subgroups: those with a low score on Item 4 (0 and 1) vs. those with a high score (2 or 3) at baseline. Then we extended *Model 1* with the previous grouping variable (including the main effect, its interaction with pandemic status, with follow-up, and a 3-way interaction with pandemic status and follow-up). This parameterization allowed us to test whether the subgroups based on Item 4 showed different behaviors before and during the pandemic.

Given that RSS has a unidimensional structure (14, 27), we decided against conducting any post-hoc analyses on RSS questions.

All statistical analyses were performed using IBM SPSS Statistics version 29.0.1.0. Statistical significance was set at 2-tailed *p* < 0.05.

## 3 Results

### 3.1 Baseline characteristics of the participants

Table 1 presents the descriptives for the study sample by sex, group status (pre-pandemic/lockdown), and timepoint (baseline/follow-up).

**Table 1:** Characteristics of pre-pandemic and lockdown classes.

	Pre-pandemic classes		Lockdown classes	
	Baseline	Follow-up	Baseline	Follow-up
Boys				
*n*	99		122	
Age, mean ± SD	16.08 ± 0.60	–	16.16 ± 0.63	–
Family structure, n (%)				
Two-parent	71 (71.7%)†	66 (66.7%)†	85 (69.7%)	83 (68.0%)
Single-parent	17 (17.2%)†	19 (19.2%)†	20 (16.4%)	21 (17.2%)
Stepfamily	11 (11.1%)†	14 (14.1%)†	17 (13.9%)	18 (14.8%)
Family communication, mean ± SD	17.53 ± 2.61†	16.44 ± 3.56†	16.97 ± 3.26	16.79 ± 3.56
Girls				
*n*	128		118	
Age, mean ± SD	15.92 ± 0.68*	-	16.14 ± 0.64*	-
Family structure, n (%)				
Two-parent	94 (73.4%)*	92 (71.9%)	78 (66.1%)*	73 (61.9%)
Single-parent	25 (19.5%)*	26 (20.3%)	19 (16.1%)*	27 (22.9%)
Stepfamily	9 (7.0%)*	10 (7.8%)	21 (17.8%)*	18 (15.3%)
Family communication, mean ± SD	17.43 ± 2.56 †	16.02 ± 4.15†	17.00 ± 3.11†	16.38 ± 3.84†

SD: standard deviation. *p < 0.05 (Baseline data of pre-pandemic vs. lockdown classes and follow-up data of pre-pandemic vs. lockdown classes were compared with Chi-squared tests for categorical variables and independent samples *t*-tests for continuous variables). ^†^*p* < 0.05 (Baseline vs. follow-up data within pre-pandemic and lockdown classes were compared with Marginal Homogeneity tests for categorical variables and paired samples *t*-tests for continuous variables).

Regarding boys, age, family structure, and family communication results were similar in the pre-pandemic and the lockdown classes at both timepoints (all *p* > 0.05).

Girls in lockdown classes were 0.22 years older compared to the pre-pandemic group. Furthermore, the baseline family structure of the pre-pandemic and lockdown classes differed significantly for girls with a higher proportion of stepfamilies in the lockdown group. However, we found no significant differences in family structure or family communication at follow-up.

### 3.2 Changes in family structure and communication over follow-up

When comparing baseline and follow-up characteristics within groups, we found that the distribution of family structure changed (less two-parent families at follow-up) and family communication worsened significantly from baseline to follow-up in the pre-pandemic group for boys. No other significant changes were found in any of the groups from baseline to follow-up (Table 1).

### 3.3 Changes in the WHO-5 well-being score

**Figure 2:**
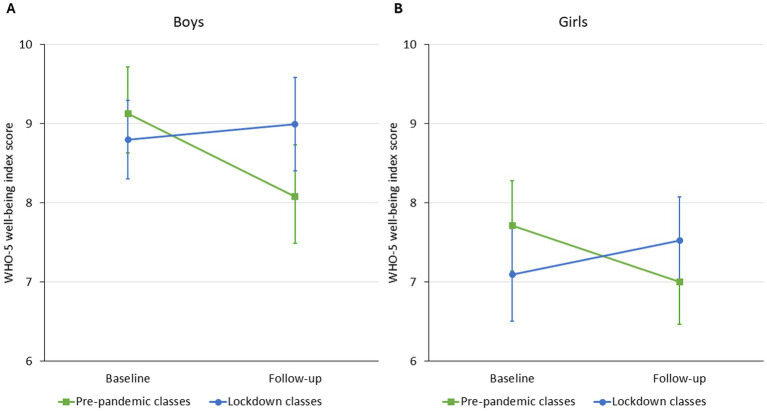
Estimated marginal means (Model 1) of boys’ (Panel A) and girls’ (Panel B) WHO-5 well-being index score of pre-pandemic and lockdown classes from baseline to follow-up.

According to the unadjusted model (*Model 1*), WHO-5 well-being scores were similar in the pre-pandemic and lockdown groups of boys at baseline. The pre-pandemic boys’ score declined by 1.05 (95% confidence intervals [CI]: −1.72 to −0.38) points over 1 year of follow-up. In contrast, the lockdown group showed a significantly smaller decline during follow-up, leading to a non-significant but positive point estimate of change. Adjustment for family structure and family communication (*Model 2*) did not materially change this pattern. Furthermore, family communication showed a significant positive association with the WHO well-being score cross-sectionally: better communication within the family was associated with higher score on well-being of boys (Table 2; Figure 2).

**Table 2:** Results of linear mixed models for WHO well-being score.

	Model 1	Model 2†		
	Estimate (95% CI)	*p*-value	Estimate (95% CI)	*p*-value
Boys				
Intercept	9.13 (8.54 to 9.72)		9.04 (8.42 to 9.66)	
Classes				
Pre-pandemic	ref.		ref.	
Lockdown	−0.33 (−1.10 to 0.45)	0.408	−0.20 (−0.96 to 0.55)	0.594
Time	−1.05 (−1.72 to − 0.38)*	**0.002**	−0.82 (−1.49 to − 0.14)*	**0.018**
Classes*Time				
Pre-pandemic	ref.		ref.	
Lockdown	**1.24 (0.35 to 2.12)**	**0.006**	**1.05 (0.17 to 1.93)**	**0.020**
Family structure				
Two-parent	–	–	ref.	
Single-parent	–	–	0.03 (−0.81 to 0.86)	0.952
Stepfamily	–	–	−0.53 (−1.46 to 0.41)	0.269
Family communication	–	–	0.82 (0.49 to 1.16)*	**<0.001**
Girls				
Intercept	7.71 (7.14 to 8.28)		7.66 (7.08 to 8.24)	
Classes				
Pre-pandemic	ref.		ref.	
Lockdown	−0.62 (−1.44 to 0.20)	0.139	−0.61 (−1.41 to 0.18)	0.131
Time	−0.71 (−1.31 to − 0.11)*	**0.022**	−0.38 (−1.01 to 0.24)	0.225
Classes*Time				
Pre-pandemic	ref.		ref.	
Lockdown	**1.14 (0.28 to 2.01)**	**0.010**	**1.03 (0.15 to 1.90)**	**0.022**
Family structure				
Two-parent	–	–	ref.	
Single-parent	–	–	−0.77 (−1.52 to − 0.01)*	**0.047**
Stepfamily	–	–	0.68 (−0.29 to 1.64)	0.167
Family communication	–	–	0.89 (0.58 to 1.21)*	**<0.001**

95% CI: 95% Confidence Interval. *p < 0.05. ^†^Adjusted for time-varying family structure and family communication. Bold values indicate significant results.

In general, girls had lower WHO-5 well-being scores than boys. The overall pattern of change in girls was similar to boys in the unadjusted model (*Model 1*): similar scores in the pre-pandemic and the lockdown groups at baseline with a significant decline in the pre-pandemic group (mean difference [MD]: 0.71, 95%CI: −1.31 to −0.11), and a significantly different change leading to a non-significant improvement in the lockdown group. After adjustment for time-varying family communication and structure (*Model 2*), the decline in the pre-pandemic group hugely attenuated and became non-significant, while the difference in the change between the groups remained leading to an actual improvement in well-being in the lockdown group (MD: 0.64, 95%CI: 0.01 to 1.72). Living in a single-parent family was associated with a worse well-being score (−0.77 [95%CI: −1.52 to −0.01]) compared to two-parent families and stepfamilies cross-sectionally. Family communication had a positive effect of on well-being with a similar effect size as it did in boys (Table 2; Figure 2).

### 3.4 Changes in the individual items of the WHO-5 well-being index during follow-up

After experiencing an unexpected improvement in well-being during the pandemic, we decided to clarify the reason by running separate models for the individual items of the WHO-5 WBI using similar linear mixed models with group status as the only predictor. When analyzing individual items of the WHO-5 WBI questionnaire, the boys’ lockdown group had slightly higher scores at baseline for Item 1 (feeling cheerful in good spirits; 0.32 [95%CI: 0.09 to 0.54]) and Item 3 (feeling active and vigorous; 0.27 [95%CI: 0.03 to 0.50]) compared to the pre-pandemic group. No significant change for the group of pre-pandemic boys was found during the follow-up period. As for the lockdown group, no change in Items 1, 2, 3, and 5 was found, however, the change over time was significantly different for Item 4 (waking up feeling fresh and relaxed) between the pre-pandemic and lockdown groups, showing an improvement in the lockdown group from baseline to follow-up (Table 3).

**Table 3:** Results of linear mixed models for WHO-5 WBI items.

	WHO 1		WHO 2		WHO 3		WHO 4		WHO 5	WBI without WHO 4^†^		
	Estimate (95% CI)	*p*-value	Estimate (95% CI)	*p*-value	Estimate (95% CI)	*p*-value	Estimate (95% CI)	*p*-value	Estimate (95% CI)	*p*-value	Estimate (95% CI)	*p*-value
Boys												
Intercept	1.89 (1.72 to 2.06)		1.66 (1.49 to 1.83)		1.68 (1.50 to 1.85)		1.15 (0.96 to 1.34)		1.57 (1.39 to 1.74)		6.76 (6.20 to 7.32)	
Classes												
Pre-pandemic	ref.		ref.		ref.		ref.		ref.		ref.	
Lockdown	0.32 (0.09 to 0.54)*	**0.006**	0.18 (−0.05 to 0.41)	0.131	0.27 (0.03 to 0.50)*	**0.028**	−0.09 (−0.33 to 0.16)	0.505	0.19 (−0.05 to 0.42)	0.119	0.98 (0.22 to 1.74)*	**0.012**
Time	0.15 (−0.05 to 0.35)	0.141	0.00 (−0.21 to 0.21)	0.985	0.15 (−0.06 to 0.36)	0.157	−0.17 (−0.39 to 0.05)	0.135	0.01 (−0.21 to 0.22)	0.937	0.34 (−0.30 to 0.98)	0.290
Classes*Time												
Pre-pandemic	ref.		ref.		ref.		ref.		ref.		ref.	
Lockdown	−0.18 (−0.45 to 0.10)	0.203	0.08 (−0.20 to 0.36)	0.555	−0.18 (−0.46 to 0.11)	0.220	0.42 (0.12 to 0.72)*	**0.007**	−0.10 (−0.39 to 0.19)	0.498	−0.40 (−1.26 to 0.46)	0.359
Girls												
Intercept	1.76 (1.62 to 1.89)		1.42 (1.28 to 1.56)		1.62 (1.47 to 1.77)		1.09 (0.95 to 1.24)		1.53 (1.38 to 1.69)		6.31 (5.81 to 6.80)	
Classes												
Pre-pandemic	ref.		ref.		ref.		ref.		ref.		ref.	
Lockdown	0.18 (−0.01 to 0.38)	0.067	−0.04 (−0.24 to 0.17)	0.723	0.03 (−0.19 to 0.25)	0.790	−0.31 (−0.53 to − 0.10)*	**0.004**	−0.19 (−0.41 to 0.03)	0.089	0.01 (−0.70 to 0.72)	0.980
Time	0.16 (−0.01 to 0.33)	0.053	−0.06 (−0.25 to 0.13)	0.546	−0.14 (−0.32 to 0.04)	0.136	−0.28 (−0.45 to − 0.12)*	**0.001**	−0.11 (−0.29 to 0.07)	0.225	−0.12 (−0.65 to 0.42)	0.665
Classes*Time												
Pre-pandemic	ref.		ref.		ref.		ref.		ref.		ref.	
Lockdown	−0.07 (−0.31 to 0.17)	0.561	0.17 (−0.11 to 0.44)	0.227	0.15 (−0.12 to 0.41)	0.272	0.41 (0.17 to 0.65)*	**<0.001**	0.20 (−0.05 to 0.46)	0.120	0.42 (−0.35 to 1.19)	0.280

95% CI: 95% Confidence Interval. *p < 0.05. ^†^Sum of Items 1, 2, 3, and 5 from WHO WBI. Bold values indicate significant results.

As for girls, baseline differences were detected only for Item 4 with lockdown girls having a lower score (−0.31 95%CI: −0.53 to −0.10). The score of Item 4 declined in pre-pandemic girls during the follow-up period, while (similarly to boys) the lockdown girls’ score changed in the opposite direction with a similar effect size observed in boys (Table 3).

### 3.5 Changes in the WHO-5 well-being score in subgroups based on item 4 at baseline

**Figure 3:**
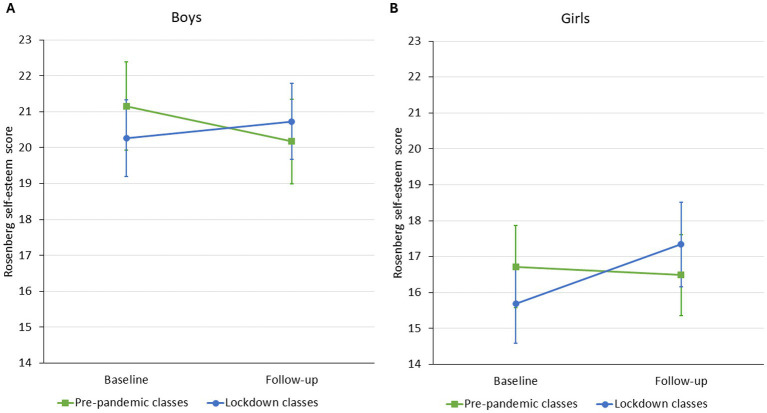
Estimated marginal means (Model 1) of boys’ (Panel A) and girls’ (Panel B) WHO-5 well-being index score by subgroups based on WHO-5 Item 4.

Since the only significant difference between the pre-pandemic and the lockdown groups was in the change in the score on Item 4 (waking up feeling fresh and rested), we hypothesized that adolescents who struggled with waking up early may have profited the most from the lockdowns. To test this, we compared the changes in the WHO-5 well-being score in subgroups based on Item 4 at baseline. The WHO-5 WBI score of boys waking up feeling fresh and relaxed (Item 4 score ≥ 2) declined similarly in the pre-pandemic and the lockdown classes (MD of changes: 0.33 [95%CI: −1.10 to 1.77]). In contrast, boys with lower scores on Item 4 at baseline behaved differently in pre-pandemic and lockdown groups: total WHO-5 WBI score of the pre-pandemic group declined, while the score of lockdown group increased (MD of changes: 1.20 [95%CI: 0.15 to 2.25]) (Figure 3).

The changes observed in girls were similar to boys. The WHO-5 WBI score of girls waking up feeling fresh and relaxed (Item 4 score ≥ 2) declined similarly in the pre-pandemic and the lockdown classes (MD of changes: -0.31 [95%CI: −2.02 to 1.41]). In contrast, girls with lower scores on Item 4 at baseline behaved differently in the pre-pandemic and the lockdown groups: total WHO-5 WBI score of the pre-pandemic group declined, while the score of the lockdown group increased (MD of changes: 1.20 [95%CI: 0.23 to 2.17]) (Figure 3).

### 3.6 Changes in the Rosenberg self-esteem scale

**Figure 4:**
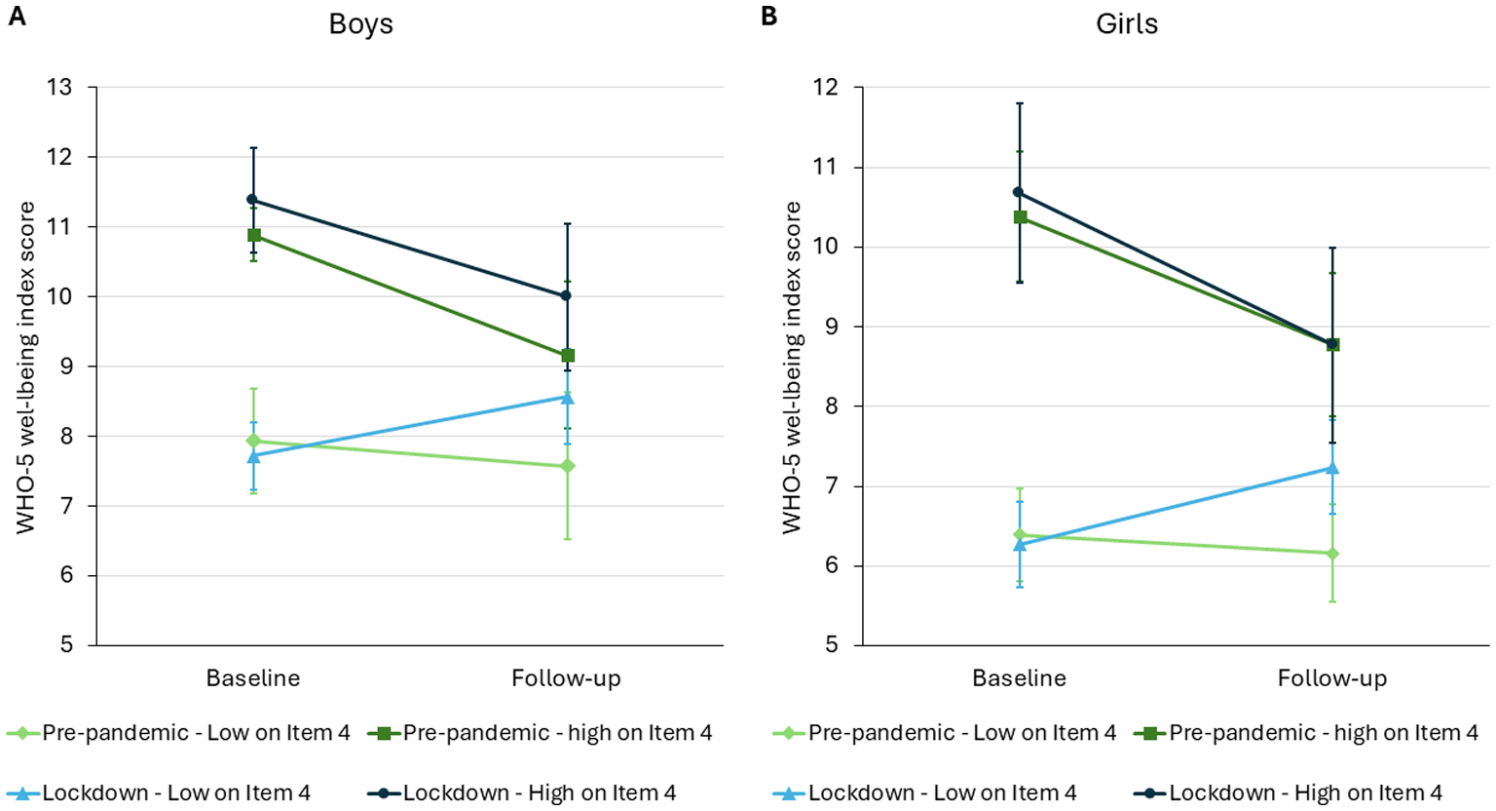
Estimated marginal means (Model 1) of boys’ (Panel A) and girls’ (Panel B) Rosenberg self-esteem score of pre-pandemic and lockdown classes from baseline to follow-up.

The average score on the Rosenberg self-esteem scale was similar for boys in the pre-pandemic and the lockdown groups without a significant change in unadjusted models (*Model 1*) during the one-year follow-up. The model adjusted for family structure and communication (*Model 2*) yielded similar results. Furthermore, this model also found that living in a stepfamily was associated with a 2.26 (95%CI: 0.35 to 4.16) point lower score on the self-esteem scale, as well as better family communication was associated with significantly higher self-esteem (Table 4; Figure 4).

**Table 4:** Results of linear mixed models for score on Rosenberg self-esteem scale.

	Model 1		Model 2^†^	
	Estimate (95% CI)	*p*-value	Estimate (95% CI)	*p*-value
Boys				
Intercept	21.16 (19.92 to 22.40)	0.284	21.09 (19.71 to 22.46)	
Classes				
Pre-pandemic	ref.		ref.	
Lockdown	−0.89 (−2.54 to 0.75)	0.284	−0.42 (−2.11 to 1.27)	0.627
Time	−0.99 (−2.13 to 0.16)	0.092	−0.33 (−1.50 to 0.85)	0.582
Classes*Time				
Pre-pandemic	ref.		ref.	
Lockdown	1.45 (−0.06 to 2.96)	0.060	0.87 (−0.62 to 2.36)	0.249
Family structure				
Two-parent	–	–	ref.	
Single-parent	–	–	−0.86 (−2.55 to 0.83)	0.318
Stepfamily	–	–	**−2.26 (−4.16 to − 0.35)**	**0.020**
Family communication	–	–	**1.15 (0.55 to 1.75)**	**<0.001**
Girls				
Intercept	16.72 (15.57 to 17.87)		16.74 (15.55 to 17.92)	
Classes				
Pre-pandemic	ref.		ref.	
Lockdown	−1.03 (−2.63 to 0.57)	0.205	−0.88 (−2.44 to 0.68)	0.269
Time	−0.23 (−1.30 to 0.83)	0.666	0.31 (−0.79 to 1.40)	0.584
Classes*Time				
Pre-pandemic	ref.		ref.	
Lockdown	**1.89 (0.42 to 3.35)**	**0.012**	**1.65 (0.17 to 3.13)**	**0.029**
Family structure				
Two-parent	–	–	ref.	
Single-parent	–	–	−0.99 (−2.57 to 0.60)	0.223
Stepfamily	–	–	−0.56 (−2.57 to 1.45)	0.583
Family communication	–	–	**1.61 (1.00 to 2.22)**	**<0.001**

95% CI: 95% Confidence Interval. *p < 0.05. ^†^Adjusted for time-varying family structure and family communication. Bold values indicate significant results.

Girls had a lower score on the Rosenberg self-esteem scale compared to boys. There was no difference in the self-esteem scores either in the unadjusted or the adjusted models between pre-pandemic and lockdown groups at baseline. We found no significant change in the self-esteem score in the pre-pandemic classes during the one-year follow-up, while the change in the lockdown classes was significantly larger, leading to an increase over time. Model 2 confirmed this finding. Furthermore, we found that better family communication was associated with higher self-esteem score in girls, while family structure was not related to self-esteem (Table 4; Figure 4).

## 4 Discussion

### 4.1 Short summary

In a secondary epidemiological analysis of an intervention program of 9^th^ grade secondary school students in a Hungarian city, we compared changes in well-being and self-esteem before and during the COVID-19 pandemic over a one-year period.

Our results showed that adolescents’ well-being declined under ordinary circumstances over 1 year, but this decline was significantly smaller (even showing positive point estimates) during the COVID-19 pandemic. This finding disproved our hypothesis. The differences between the changes in the pre-pandemic vs. the lockdown cohorts remained significant even after adjusting for family communication and structure. When we looked for a potential explanation of this observation, we found that 4 of the 5 WHO-5 WBI items changed similarly before and during the pandemic, while there was a significant difference in the change of Item 4 (waking up feeling fresh and relaxed) between the cohorts over follow-up. Based on this, we tested for an interaction between item 4 scores at baseline and lockdown status over time. This analysis showed that the total WHO-5 WBI score declined in those with a high baseline score on item 4 irrespective of sex and lockdown status over time. For those with a low baseline score on item 4, it remained stable in those not affected by the lockdown, while it improved significantly in those adolescents affected by the pandemic.

While the self-esteem of boys changed similarly in the cohorts over follow-up, self-esteem of girls affected by the pandemic increased compared to the pre-pandemic classes over follow-up. Again, adjustment for family structure and communication had no major effect on these observations.

Finally, our study also revealed that better family communication was consistently associated with higher well-being and higher self-esteem for both boys and girls.

### 4.2 Results in context of the literature

#### 4.2.1 Sex differences in well-being and self-esteem

Our study showed that girls overall had a lower well-being and self-esteem than boys, which correlates with the literature (28–30). Girls tend to have more depressive symptoms and exhibit stronger anxiety than boys, which may translate to lower well-being (31, 32). As for self-esteem, girls tend to evaluate their own physical appearance and intellectual abilities more negatively than boys, potentially explaining why girls have a lower self-esteem in adolescence than boys (33). Furthermore, the observed sex differences may be related to sex-related variations in changes related to puberty or differences in the roles of sexes, their responsibilities, and the support system (31).

#### 4.2.2 Changes in well-being associated with the pandemic

Our study revealed a decrease in well-being of pre-pandemic adolescents over a one-year period, which is in line with the results of the latest Health Behavior in School-aged Children Study of the WHO (28). Surprisingly, this decline was not observed in the pandemic cohort despite several studies demonstrating negative effects of school lockdowns on mental health (34–37). According to our post-hoc analysis, changes in 4 of the 5 items of the WHO-5 WBI were similar before and during the pandemic, while Item 4 that investigates feelings after waking up, sleeping more and feeling more rested decreased significantly less during the pandemic. Based on these findings we suspect that the overall change in well-being is linked to more sleep time and/or later wake-up time among those students that had a low response on Item 4 at baseline, as students attended class from home and did not have to commute to school in the early hours (38, 39). A study performed in Austrian supports our findings, as it also revealed an improvement in the WHO-5 WBI score during remote schooling compared to a period when schools were reopened. However, they did not look for an explanation of this counterintuitive observation (40). Furthermore, a qualitative study on Scottish adolescents claimed the disruption to schooling had positive impact on their mental health and well-being due to reduced school-related difficulties and workload (41).

#### 4.2.3 Changes in self-esteem during the pandemic

In our study, self-esteem remained broadly stable in in the pre-pandemic cohorts over follow-up, which is in line with the results of other studies (42, 43). In contrast, girls’ self-esteem improved during the pandemic compared to the pre-pandemic group. Unlike our finding, other studies reported no change (44) or a slight decrease (15) in self-esteem, however the latter used different instrument. Furthermore, studies on the effect of COVID-19 school lockdowns on self-esteem are scarce. Self-esteem reflects one’s perception of their own worth and value (14) and is affected by several factors among which school expectations and academic achievement seem to play an important role (30, 45, 46). Since girls are more concerned about academic achievement (47), lower academic expectations may explain the observed improvement of the girls’ self-esteem during the pandemic.

#### 4.2.4 Cross-sectional determinants of well-being and self-esteem

Better family communication was cross-sectionally related to higher well-being and self-esteem both in boys and girls in our study that well corresponds to the literature (48, 49). In addition family communication has a mediating effect between family resilience and family functioning (50) and could also lead to better mental health of adolescents (51). Our results showed that both boys and girls with better family communication independent of the presence of adversities, such as the COVID-19 pandemic, as better family communication incorporating elements of approach coaching may decrease distress (23). Approach coaching is defined as “any behavioral, cognitive, or emotional activity that is directed toward a threat (e.g., problem solving or seeking information)” (52) and is in contrast to avoidance of the situation, which is linked to an increase of distress in time of adversities (23).

### 4.3 Strengths and limitations

Our study benefits from several strengths. First, our participants from a deprived Central-European region well represent adolescents from similar circumstances giving some external validity to our findings. Given that we collected data on within person changes over time, we have sufficient power to investigate relatively small absolute changes in well-being and self-esteem. Furthermore, the fact that the pre-pandemic and the lockdown cohorts came from the same source population and had similar baseline characteristics, the bias resulting from cohort effect may be minimal. The longitudinal design of our study is also notable because most studies investigating similar questions had a cross-sectional design or did not have adequate control groups.

Our study has some limitations that has to be acknowledged. First, neither the participation rate, nor the capture of participants at follow-up were perfect that limits external validity. Similarly, our participants represent an ethnically homogenous group, thus extrapolation to non-white adolescents is limited. Our outcomes are based on short versions of the well-being and the self-esteem questionnaires that may limit their sensitivity. Furthermore, the WHO-5 questionnaire may be prone to an ‘influential question’: given that students were not required to get up early during the pandemic, Item 4 of the WHO-5 WBI questionnaire showed a significant improvement. However, we think that this does not necessarily reflect an improvement in overall well-being. This observation highlights the importance to investigate the effect of an intervention or changing circumstance on each item of the questionnaire and shows limited generalizability of this measure. Furthermore, even though the WHO-5 WBI is widely used to measure well-being in different phases of life, the definition of well-being and the factors affecting it may differ between adults and adolescents (53). For instance, while the WHO-5 well-being index was shown to be associated with psychosocial working factors among adults (54), its association with school environment or academic pressure have not been investigated as extensively, which factors can be relevant regarding the well-being of adolescents. Moreover, our questionnaire did not include questions and instruments that measured certain feelings related to the pandemic either, such as fear and anxiety of the pandemic (55), that may also have a great impact on the well-being of adolescents. The main reason for this is that our questionnaire was developed before the pandemic. Similarly, other determinants of well-being and self-esteem (such as, social connections (56), teacher-student relationship (57), or environmental factors (56)) were not captured in our study, and these and other unmeasured confounders could have biased our findings. Given this and the fact that ours is a *post hoc* analysis of health promotion program precludes determining causal relationships and our results are only for hypothesis generation.

## 5 Conclusion

In conclusion, our study shows that adolescents’ well-being and self-esteem changed differently during the COVID-19 lockdowns compared to the pre-pandemic period. While it is expected that the fear of an unknown pandemic and social distancing can raise anxiety and depression in populations, certain aspects of life may unexpectedly improve. For instance, our study showed that adolescents felt more rested during the lockdown period of the pandemic that had a positive effect on their WHO-5 well-being scores. Similarly, we found a robust improvement in the self-esteem of girls during the pandemic potentially related to a less intensive feedback from peers and teachers. If our results reflect a causal relationship between longer sleep and well-being, schools could consider starting teaching at later hours for adolescents.

In addition to this, our results strongly support the beneficial role of good family communication in adolescents’ well-being and self-esteem, as well as other mental health issues.

Another important conclusion relates to the limitation of general well-being questionnaires and especially short versions of them in measuring well-being in special circumstances. We think that these questionnaires should be supplemented with other instruments that evaluate other aspects of well-being and also aspects of the circumstances we intend to investigate.

## Data Availability

The raw data supporting the conclusions of this article will be made available by the authors, without undue reservation.
